# Melatonin enhances DNA repair capacity possibly by affecting genes involved in DNA damage responsive pathways

**DOI:** 10.1186/1471-2121-14-1

**Published:** 2013-01-07

**Authors:** Ran Liu, Alan Fu, Aaron E Hoffman, Tongzhang Zheng, Yong Zhu

**Affiliations:** 1 Key Laboratory of Environmental Medicine Engineering, Ministry of Education, School of Public Health, Southeast University, Nanjing, 210009, China; 2 Department of Environmental Health and Sciences, Yale School of Public Health, New Haven, Connecticut, CT, 06520, USA; 3 Department of Epidemiology, Tulane School of Public Health and Tropical Medicine and Tulane Cancer Center, New Orleans, LA, 70112, USA

**Keywords:** Melatonin, DNA repair, Comet assay, Genome-wide expression, Network analysis

## Abstract

**Background:**

Melatonin, a hormone-like substance involved in the regulation of the circadian rhythm, has been demonstrated to protect cells against oxidative DNA damage and to inhibit tumorigenesis.

**Results:**

In the current study, we investigated the effect of melatonin on DNA strand breaks using the alkaline DNA comet assay in breast cancer (MCF-7) and colon cancer (HCT-15) cell lines. Our results demonstrated that cells pretreated with melatonin had significantly shorter Olive tail moments compared to non-melatonin treated cells upon mutagen (methyl methanesulfonate, MMS) exposure, indicating an increased DNA repair capacity after melatonin treatment. We further examined the genome-wide gene expression in melatonin pretreated MCF-7 cells upon carcinogen exposure and detected altered expression of many genes involved in multiple DNA damage responsive pathways. Genes exhibiting altered expression were further analyzed for functional interrelatedness using network- and pathway-based bioinformatics analysis. The top functional network was defined as having relevance for “DNA Replication, Recombination, and Repair, Gene Expression, [and] Cancer”.

**Conclusions:**

These findings suggest that melatonin may enhance DNA repair capacity by affecting several key genes involved in DNA damage responsive pathways.

## Background

Melatonin (MLT), a hormone secreted by the pineal gland, plays an important role not only in the regulation of the circadian rhythm but also in the modulation of cancer risk by acting as an anti-inflammatory agent and an antioxidant (reviewed in [1,2]). Recent epidemiologic evidence have pointed to an inverse relationship between night shift work and melatonin levels [3], which provides a possible explanation for the observation of increased breast cancer risk among female shift workers [4,5]. By the same token, significantly decreased plasma melatonin levels have been found in patients with endometrial cancer [6,7]. In animal studies, melatonin has been shown to inhibit the development of spontaneous and chemically induced mammary carcinogenesis [8], colon and hepato-carcinogenesis, [9], and carcinogenesis of soft tissue, the lung, the uterine cervix and the vagina [10-13].

*In vitro* functional analyses using cancer cell lines such as MCF-7 have been performed to investigate the effects of melatonin on cell growth, DNA synthesis, cell cycle, and apoptosis [14-17]. These studies have shown that melatonin may reduce MCF-7 cell proliferation by modulating cell-cycle kinetics, DNA synthesis, and apoptotic processes. Other studies have further demonstrated that melatonin can inhibit fatty acid growth-factor uptake [18] and telomerase activity in cancer cells [19]. The role of melatonin in DNA repair pathways was also recently documented in a study that showed a protective role for melatonin against oxidative DNA damage [20].

However, the impact of melatonin on DNA repair capacity for DNA strand breaks, the most common type of DNA damage, has not been previously examined using the comet assay, a reliable method for detecting and measuring DNA damage and DNA repair capacity [21]. Moreover, little is known about how melatonin affects expression of the genes involved in DNA damage response. In the current study, we performed both a comet assay and genome-wide expression assay using breast cancer cells (MCF-7) and colon cancer cells (HCT-15) to investigate the impact of physiological concentrations of melatonin on DNA repair capacity and expression of genes in DNA damage responsive pathways.

## Methods

### Cell culture and treatments

MCF-7, a human breast cancer cell line, and HCT-15, a human colon cancer cell line, were purchased from the American Type Culture Collection (ATCC, Manassas, VA ). MCF-7 cells were maintained as monolayer cultures in 25 cm^2^ polystyrene culture flasks (Falcon, Becton Dickinson BioScience, Le Pont de Claix, France) in Dulbecco's Modified Eagle's Medium (DMEM) supplemented with 10% fetal bovine serum, 1% penicillin-streptomycin (Invitrogen, Carlsbad, CA) and 0.01mg/ml insulin (Sigma-Aldrich, St. Louis, MO). HCT-15 cells were maintained in Roswell Park Memorial Institute (RPMI) 1640 media supplemented with 10% fetal bovine serum and 1% penicillin-streptomycin. Cells were incubated at 37°C in a humid atmosphere containing 5% CO_2_ and subcultured every 3~4 days.

Before each experiment, stock subconfluent monolayers of MCF-7 or HCT-15 cells were incubated with 0.25% trypsin-EDTA at 37°C for 3~5 min. MCF-7 cells were then resuspended in DMEM supplemented with 10% FBS and 0.01mg/ml insulin and HCT-15 cells were resuspended in RPMI 1640 media supplemented with 10% fetal bovine serum. Cell number and viability were determined by staining a small volume of cell suspension with 0.4% trypan blue saline solution and examining the cells in a hemocytometer. For all assays, MCF-7 cells were seeded at a density of 3~5×10^5^ cells/ml in DMEM supplemented with 10% FBS, 0.01mg/ml insulin, and 1% penicillin-streptomycin for 24 hr (37°C, 5% CO_2_), and HCT-15 cells were seeded in RPMI 1640 supplemented with 10% FBS. After 24 hours, the media were replaced with fresh media containing either 1 nM melatonin (Sigma Chemical Co., St Louis, MO, USA), or the diluent (ethanol, final concentration 0.000005%) and cells were incubated for another 24 hours. Methyl methanesulfonate (MMS, mutagenic agent) (0.0125%, v/v, Sigma Chemical Co., St Louis, MO, USA) or PBS was added to the culture medium for an 1-hour treatment followed by an extra 3-hour recovery time in fresh medium. Cells were then harvested for the comet assay and RNA extraction.

### Comet assay

DNA damage was assessed through the use of a comet assay that was first described by Östling & Johanson [22] and later improved by Singh et al. [23]. Following MLT and MMS/PBS treatment as described above, cells were harvested and mixed with 0.5% low-melting agarose [24] and fixed onto frosted slides pre-coated with 1.0% normal melting agarose (NMA). The slides were immersed in cold, freshly made lysing solution in the dark at 4°C for at least 1 hr and then rinsed with neutralization buffer to remove detergents and salts. Slides were then placed in a horizontal electrophoresis device at 4°C in alkaline buffer (pH>13) for 30 min to allow for DNA unwinding and expression of alkali-labile damage, followed by electrophoresis for 30 min (25V, 300mA), which allows damaged and/or broken DNA to migrate away from the nucleus. After electrophoresis, the slides were washed three times with a neutralization buffer. Slides were then stained with ethidium bromide (10 μg/ml), visualized by fluorescence microscopy, and analyzed using the Komet 5.0 comet assay analysis software which quantitatively determines the extent of DNA damage in each sample using the mean Olive tail moment calculation as previously described [25]. The head of the comet image consists of intact DNA, while the tail material is comprised of damaged DNA. Longer “tails” are associated with a greater accumulation of damage. Olive tail moment is defined as the function of the tail length and the precentage of DNA in the tail. Olive tail moment = (Tail.mean-Head.mean) X Tail%DNA/100. The mean Olive tail moment was determined for 200 cells (two slides with 100 cells per slide) from each of the four treatment groups (MLT, MLT+MMS, MMS, and Control) and all pairwise comparisons were examined using Fisher’s PLSD.

### Genome-wide expression microarray analysis

Genome-wide expression array analysis was only performed following MLT+MMS and MMS treatments. MCF-7 cells were harvested and total RNA was isolated using the RNeasy Mini Kit (Qiagen, Valencia, CA), with on-column DNA digestion, according to the manufacturer’s instructions for mammalian cells. Genome-wide expression differences among MLT/MMS and diluents/MMS treated cells were determined by using the HumanHT-12 v4 Expression BeadChip, Illumina's latest whole-genome expression array system (Illumina, San Diego, CA). Samples were run in biological duplicate. Array hybridization and signal scans were performed by Yale University’s W.M. Keck Foundation Biotechnology Research Laboratory. The images were analyzed using the Beadstudio software. Quality control and data analysis were carried out according to the instructions provided by Illumina. All array data have been uploaded to the Gene Expression Omnibus (GEO) database, and can be accessed via their website (http://www.ncbi.nlm.nih.gov/geo/; accession number pending).

### Pathway-based network analysis

The set of differentially expressed transcripts was investigated for network and functional interrelatedness using the Ingenuity Pathway Analysis (IPA) software tool. Fold changes in gene expression were determined with the normalized signal intensities of probes from MLT+MMS treatment divided by the signal intensities from MMS treatment. The set of differentially expressed genes and their fold change values were inputted into the IPA program and used to identify interaction models of genes using the Ingenuity Knowledge Base, a manually curated database of functional interactions extracted from peer-reviewed publications (http://www.ingenuity.com). A Fisher’s exact test based on the hypergeometric distribution was then done for each identified network to determine the likelihood of obtaining at least the same number of interrelated molecules by chance.

### Statistical analysis

Statistical analysis of DNA damage repair capacity was performed using the Komet 5.0 comet assay analysis software. Differences in DNA repair were determined for all pairwise associations of the four treatment groups (MLT, MLT+MMS, MMS, and Control) using ANOVA Fisher’s PLSD for the mean Olive tail moment with the SAS statistical software package, version 9.1 (SAS Institute, Cary, NC). The Student’s t-test was performed to compare gene expression levels using normalized expression values from the Illumina expression array for the MLT+MMS and MMS treatment groups. Transcripts were identified as significantly influenced by MLT treatment if their expression differences fit the criteria of *p*<0.05.

## Results

**Figure 1:**
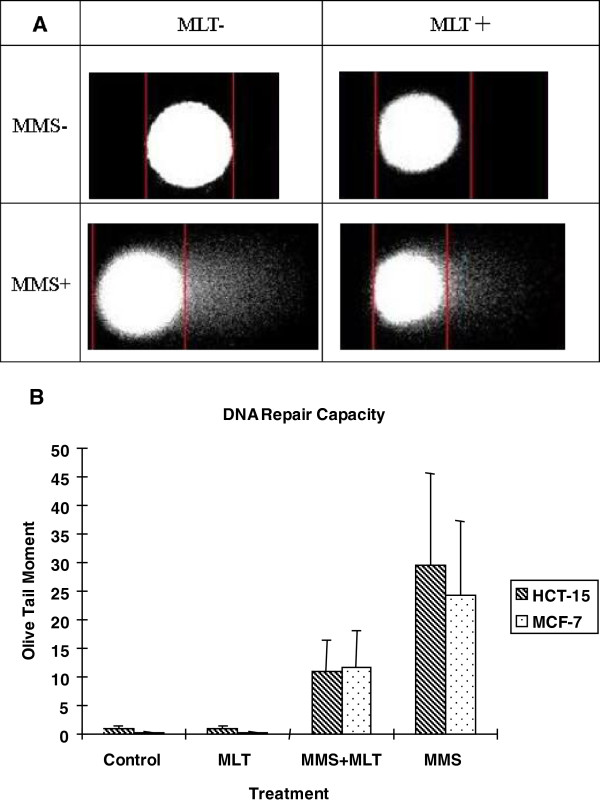
**DNA damage repair capacity as assessed by the Comet Assay. **(**A**) Representative cell images from each of the four treatment groups using MCF-7 cells. (**B**) Mean Olive tail moment determined from each of the four treatment groups using HCT-15 and MCF-7 cells. Among the samples not treated with mutagen, the difference in tail moment between cells in the control group and MLT-treated group was not significant in both cell lines (*p*=0.976 for MCF-7, *p*=0.928 for HCT-15). However, among the mutagen-treated populations, cells with MLT pretreatment had a significantly shorter mean Olive tail moment (*p*<0.0001 in both cell lines) compared to cells without MLT pretreatment. The error bar corresponds to mean±SD.

To study the impact of melatonin on DNA repair capacity for MMS-induced strand breaks, the comet assay was used to assess the differences in DNA damage accumulation and repair capacity among four treatment groups (MLT, MLT+MMS, MMS, and Control). Figure 1A shows a representative comet assay image from each treatment group using MCF-7 cells, and Figure 1B depicts the mean Olive tail moment for each group in MCF-7 and HCT-15 cells. For MCF-7 cells, the mean Olive tail moments were 0.18±0.10 for the control group, 0.22±0.10 for the MLT treatment group, 11.66±6.51 for MLT+MMS, and 24.13±13.12 for MMS only. For HCT-15, the Olive tail moments were 0.97±0.52 for the control group, 0.86±0.50 for the MLT treatment group, 10.80±5.65 for MLT+MMS, and 29.51±16.10 for MMS only.

Among the samples not treated with mutagen, the differences in mean Olive tail moments between cells in the control group and those in the MLT treatment group were not statistically significant for either cell line (*p*=0.976 for MCF-7, *p*=0.928 for HCT-15), indicating that without inducing damage, there is no difference in detected DNA damage levels. However, among the mutagen-treated populations, cells with MLT pretreatment had a significantly shorter mean Olive tail moment (*p*<0.0001 in both cell lines), as depicted in Figure 1B, indicating increased DNA repair capacity compared to cells without MLT pretreatment.

**Figure 2:**
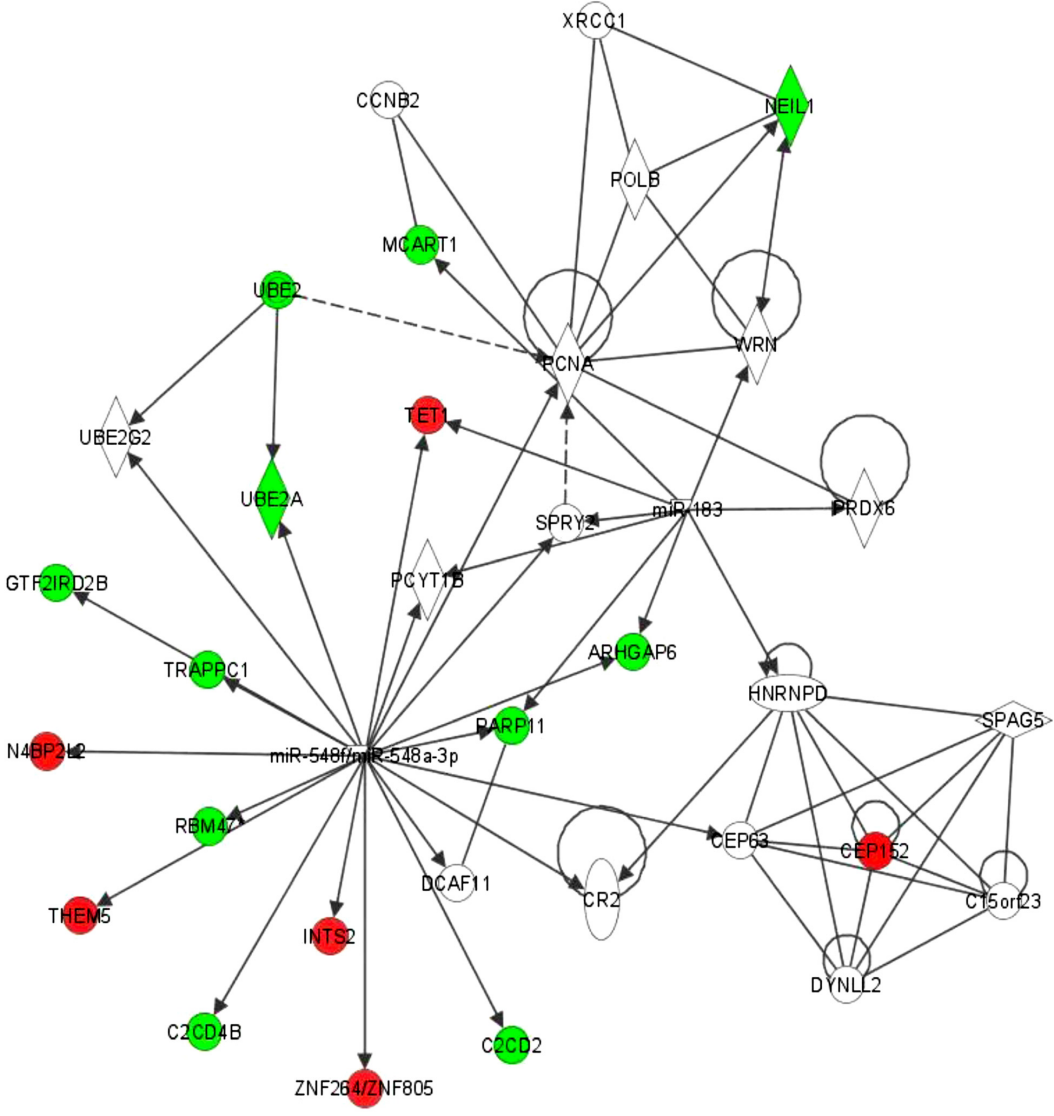
**The top network, “DNA Replication, Recombination, and Repair, Gene Expression, Cancer”, formed by genes exhibiting altered expression in MLT-pretreated MCF-7 cells upon MMS exposure relative to untreated MMS-exposed cells.** Upregulated transcripts are shaded in red, while downregulated transcripts are shaded in green, with color intensity signifying the magnitude of fold change. Each interaction is supported by at least one literature reference identified in the Ingenuity Pathway Knowledge Base, with solid lines representing direct interactions, and dashed lines representing indirect interactions.

To further investigate possible mechanisms of the observed protective effect of MLT on DNA repair capacity induced by mutagen exposure, we performed a genome-wide expression microarray analysis. The identified transcripts affected by MLT upon MMS exposure were investigated for functional interrelatedness using the IPA software. There were 104 genes identified from the expression microarray analysis, as shown in Additional file 1: Table S1. A list of the top 5 networks significantly associated with the set of input genes is displayed in Table 1. The network most significantly associated with the set of altered genes was defined as having relevance for “DNA Replication, Recombination, and Repair, Gene Expression, [and] Cancer” (Score=34, Figure 2).

**Table 1:** Functions and molecules in the top 5 networks of identified genes regulated by MLT

**Network**	**Score**	**Focus molecules**	**Top functions**	**Molecules in network**
I	34	16	DNA Replication, Recombination, and Repair, Gene Expression, Cancer	ARHGAP6, C15orf23, C2CD2, C2CD4B, CCNB2, CEP63, CEP152, CR2, DCAF11, DYNLL2, GTF2IRD2B, HNRNPD, INTS2, MCART1, miR-183, miR-548f/miR-548a-3p, N4BP2L2, NEIL1, PARP11, PCNA, PCYT1B, POLB, PRDX6, RBM47*, SPAG5, SPRY2, TET1, THEM5, TRAPPC1, UBE2, UBE2A, UBE2G2, WRN, XRCC1, ZNF264/ZNF805
II	31	15	Cell-to-Cell Signaling and Interaction, Cellular Movement, Tissue Development	ADARB1, APPBP2, ASAP2, CADM1, CRYBA2, DENND1A, FAM96A, FRK, GLDN, KIAA0907, LYPD1, MAP3K13, miR-450, miR-873, miR-450b-5p, miR-520d-5p/miR-524-5p, miR-548p, MUL1, NFASC, NPTN, PCSK5, PDE6D, RAD54B, RBM47*, SDHAF2, SHB, SLC16A9, STK10, TBL2, TCEB3, TET1, THSD7A, TMOD2, UBE2Q2, ZNF430
III	28	14	Cell-mediated Immune Response, Cellular Movement, Hematological System Development and Function	Acan, ALOX12, ANKLE2, C2orf27A/C2orf27B, CCDC90A, CDC42, CDK5RAP2, CILP2, CLEC2D, CMA1, CST3, CTSH, EIF5A, FN1, HP, IFI27, IL13, IL11RA, ITGAE, LLGL2, miR-29b/miR-29c/miR-29a, PLUNC, RABGGTB, RCC2, RPL5, RPL23, SFTPB, SLC23A2, SOCS5, SPAG4, STAT3, TGFB1, tretinoin, WDR92
IV	28	14	Lipid Metabolism, Small Molecule Biochemistry, Cardiovascular System Development and Function	ATP, C17orf90, C1orf123, Ca2+, CCDC90B, DCAF11, DMC1, FPGS, FXYD6, G protein alphai, GABRP1, GBP3, GPSM1, GRB14, GTP, HIST1H2AH, HIST2H2AA3/HIST2H2AA4, HNF4A, miR-125a-3p, miR-1267/miR-582-3p, MRPS12, NOP16, P2RX2, P2RX3, P2RX5, P2RX6, PLCB3, PRDX5, PTK6, RGL2, RPL30, RPL18A, RTCD1, TAC3, THAP4
V	23	12	Cell Death, Cellular Development, Cellular Growth and Proliferation,	26s Proteasome, Akt, BEX2, BRAP, CLIP2, CXCL3, DYRK1B, ERBB3, ERK1/2, FER, HERP, HEY1, HRAS, IDE, IL17R, IL36A, Insulin, LDL, Mapk, miR-205, NFkB, Oas1h, Olfr1508, P38 MAPK, Pdgf, PDPK1, PI3K, Pvr, Rasgrf, RGS3, RPS6KA, SIRT4, TUBB, UTS2

This network consists of 36 genes, 16 of which exhibited altered expression following MLT treatment (*ARHGAP6, C2CD2, C2CD4B, CEP152, GTF2IRD2B, INTS2, MCART1, N4BP2L2, NEIL1, PARP11, RBM47, TET1, THEM5, TRAPPC1, UBE2A and ZNF264*/*ZNF805*). Among these genes are several with known roles in the regulation of DNA repair activity, including *CEP152*, a regulator of genomic integrity and cellular response to DNA damage through the ATR-mediated checkpoint signaling pathway [26]. Our results indicate that *CEP152* expression was significantly upregulated following MLT treatment. Similarly, *N4BP2L2* was induced after MMS exposure and higher *N4BP2L2* expression was detected in MLT pretreated cells upon MMS exposure. *N4BP2L2*, which encodes a phosphonoformate immuno-associated protein, is phosphorylated by ATM or ATR upon DNA damage.

**Figure 3:**
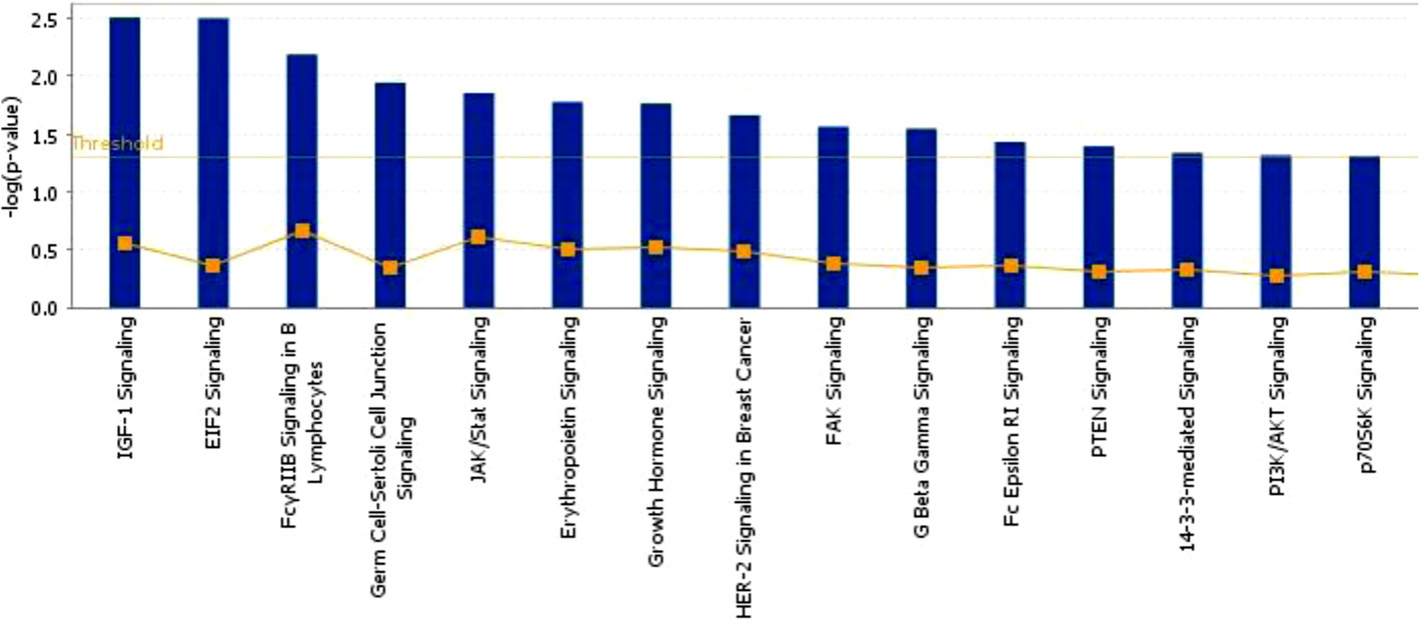
**Pathways related to genes affected by MLT treatment.** These pathways were identified by the Ingenuity Pathway Analysis software as being significantly associated with the set of genes with altered expression as a result of MLT treatment.

Many other pathways relevant to DNA repair were also identified. As illustrated in Figure 3, genes directly or indirectly influenced by MLT are involved in multiple signaling pathways associated with DNA repair, including IGF1 signaling, EIF2 signaling, JAK/Stat signaling, PI3K/AKT signaling, and P70S6K signaling.

## Discussion

The protective role of melatonin in DNA damage response observed in our study is congruent with previous findings. Since melatonin was initially identified as a free radical scavenger in 1993, many studies have shown that melatonin may protect DNA against free radical damage [27-31] by stimulating antioxidative enzymes and scavenging ·OH radicals [32,33]. In addition to stimulating DNA repair capacity, melatonin may aid in inactivating the DNA-damaging agent [34]. However, most previous studies have focused their investigation on the role of melatonin in protecting the cell against oxidative DNA damage. Our results demonstrate for the first time that melatonin may also increase DNA repair capacity against strand breaks caused by the DNA damaging agent MMS *in vitro*, as measured by the comet assay.

Although increasing evidence suggests a protective role of melatonin in the DNA repair pathway, the molecular mechanism of melatonin is still unclear, especially with regard to the potential impact of melatonin on the expression of DNA damage responsive genes. Our current study examined genome-wide gene expression affected by MLT upon MMS-induced DNA damage and found that melatonin can affect multiple DNA damage responsive pathways that are involved in several signaling pathways associated with DNA damage.

Upregulation of *CEP152* and *N4BP2L2* associated with MLT pre-treatment in our study showed positive activation of DNA repair capacity. CEP152 is a centrosomal protein involved in the maintenance of genomic integrity and response to DNA damage, acting as a scaffold facilitating the recruitment of PLK4 and CPAP to the centrosome to ensure a faithful centrosome duplication process [24,34]. Increased CEP152 expression may facilitate DNA damage checkpoint control and ATR/ATM-mediated phosphorylation in response to accumulation of DNA damage [26,35,36].

It has been suggested that N4BP2L2 is phosphorylated at serine 199 as a substrate of the protein kinases ATM and ATR in response to DNA damage [37]. ATR is known to be involved mainly in responses to DNA single-strand breaks (SSBs), while ATM is activated in response to DNA double-strand breaks (DSBs). Since ATM and ATR both trigger an overlapping set of cellular responses that promote cell cycle arrest and DNA repair [38], it is suggested that MLT may activate multiple DNA repair processes through the increase of N4BP2L2 expression and induction of ATR/ATM-mediated damage responsive pathways.

While our data support melatonin's protective role in DNA strand breaks and illuminate potential molecular pathways by which this role is fulfilled, without the inclusion of additional experimental evidence, our conclusions remain quite exploratory. Future investigations should therefore make use of alternative strand break visualization technologies, including phospho-H2AX labeling, to provide confirmational evidence, assess the kinetics of MLT's effect on DNA repair, and measure the response in specific biological pathways to MLT induction at the protein level.

## Conclusion

Our data support the hypothesis that melatonin may enhance DNA repair capacity and play a protective role in cancer development. Although the molecular mechanisms of melatonin in cancer-related biological pathways are still largely unknown, our data suggest that melatonin may participate in the regulation of several key genes involved in DNA damage repair pathways.

## Competing interests

The authors declare that they have no competing interests.

## Authors’ contribution

AF and AEH helped with statistical analysis and data interpretation. TZ and YZ helped design the study. RL performed the experiments and drafted the manuscript. All authors read and approved the final manuscript.

## Supplementary Material

Additional file 1 **Table S1.** Identified genes affected by melatonin upon MMS-induced DNA damage. Click here for file
