# Supplementary material for: Melatonin enhances DNA repair capacity possibly by affecting genes involved in DNA damage responsive pathways
Source: BMC Cell Biol. 2013 Jan 7;14:1. doi: 10.1186/1471-2121-14-1 (PMC3543845; doi:10.1186/1471-2121-14-1)
Supplement: Additional file 1 — Table S1. Identified genes affected by melatonin upon MMS-induced DNA damage. [file 1471-2121-14-1-S1.doc]

**Supplementary Table 1. Identified genes affected by melatonin upon MMS-induced DNA damage**

| **GenBank accession code** | **Gene symbol** | **Gene name** | **Fold change** | ***P* value** |
| --- | --- | --- | --- | --- |
| NM_006380.2 | *APPBP2* | Homo sapiens amyloid beta precursor protein (cytoplasmic tail) binding protein 2 (APPBP2), mRNA. | 1.05 | 0.018 |
| NM_001174.2 | *ARHGAP6* | Homo sapiens Rho GTPase activating protein 6 (ARHGAP6), transcript variant 2, mRNA. | 0.93 | 0.016 |
| NM_003887.2 | *ASAP2* | Homo sapiens ArfGAP with SH3 domain, ankyrin repeat and PH domain 2 (ASAP2), transcript variant 1, mRNA. | 0.85 | 0.036 |
| NM_032621.2 | *BEX2* | Homo sapiens brain expressed X-linked 2 (BEX2), mRNA. | 0.97 | 0.046 |
| NM_001039842.1 | *C17ORF90* | Homo sapiens chromosome 17 open reading frame 90 (C17orf90), mRNA. | 0.85 | 0.008 |
| NM_017887.1 | *C1ORF123* | Homo sapiens chromosome 1 open reading frame 123 (C1orf123), mRNA. | 0.89 | 0.018 |
| NM_199050.1 | *C2CD2* | Homo sapiens C2 calcium-dependent domain containing 2 (C2CD2), transcript variant 1, mRNA. | 0.89 | 0.033 |
| NM_214461.1 | *C2ORF27B* | Homo sapiens chromosome 2 open reading frame 27B (C2orf27B), mRNA. | 1.15 | 0.039 |
| NM_022102.1 | *C6ORF79* | Homo sapiens chromosome 6 open reading frame 79 (C6orf79), transcript variant 2, mRNA. | 1.05 | 0.032 |
| NM_021825.3 | *CCDC90B* | Homo sapiens coiled-coil domain containing 90B (CCDC90B), mRNA. | 0.91 | 0.049 |
| NM_001011649.1 | *CDK5RAP2* | Homo sapiens CDK5 regulatory subunit associated protein 2 (CDK5RAP2), transcript variant 1, mRNA. | 0.93 | 0.039 |
| NM_014985.2 | *CEP152* | Homo sapiens centrosomal protein 152kDa (CEP152), mRNA. | 1.13 | 0.030 |
| NM_153221.1 | *CILP2* | Homo sapiens cartilage intermediate layer protein 2 (CILP2), mRNA. | 0.96 | 0.043 |
| NM_199185.1 | *CLEC2D* | Homo sapiens C-type lectin domain family 2, member D (CLEC2D), transcript variant 1, mRNA. | 0.88 | 0.041 |
| NM_003388.3 | *CLIP2* | Homo sapiens CAP-GLY domain containing linker protein 2 (CLIP2), transcript variant 1, mRNA. | 0.93 | 0.019 |
| NM_057093.1 | *CRYBA2* | Homo sapiens crystallin, beta A2 (CRYBA2), transcript variant 2, mRNA. | 1.07 | 0.018 |
| NM_004390.2 | *CTSH* | Homo sapiens cathepsin H (CTSH), transcript variant 1, mRNA. | 0.96 | 0.017 |
| NM_002090.2 | *CXCL3* | Homo sapiens chemokine (C-X-C motif) ligand 3 (CXCL3), mRNA. | 1.09 | 0.017 |
| NM_024820.2 | *DENND1A* | Homo sapiens DENN/MADD domain containing 1A (DENND1A), transcript variant 2, mRNA. | 0.85 | 0.048 |
| NM_007068.2 | *DMC1* | Homo sapiens DMC1 dosage suppressor of mck1 homolog, meiosis-specific homologous recombination (yeast) (DMC1), mRNA. | 1.26 | 0.038 |
| NM_006484.1 | *DYRK1B* | Homo sapiens dual-specificity tyrosine-(Y)-phosphorylation regulated kinase 1B (DYRK1B), transcript variant c, mRNA. | 1.13 | 0.001 |
| NM_001982.2 | *ERBB3* | Homo sapiens v-erb-b2 erythroblastic leukemia viral oncogene homolog 3 (avian) (ERBB3), transcript variant 1, mRNA. | 0.90 | 0.049 |
| NM_001014812.1 | *FAM96A* | Homo sapiens family with sequence similarity 96, member A (FAM96A), transcript variant 2, mRNA. | 1.20 | 0.050 |
| NR_015410.1 | *FLJ22536* | Homo sapiens hypothetical locus LOC401237 (FLJ22536), non-coding RNA. | 1.06 | 0.028 |
| NM_004957.4 | *FPGS* | Homo sapiens folylpolyglutamate synthase (FPGS), nuclear gene encoding mitochondrial protein, transcript variant 2, mRNA. | 1.09 | 0.015 |
| NM_018284.1 | *GBP3* | Homo sapiens guanylate binding protein 3 (GBP3), mRNA. | 1.04 | 0.033 |
| NM_181789.1 | *GLDN* | Homo sapiens gliomedin (GLDN), mRNA. | 0.86 | 0.037 |
| XM_939186.1 | *GPSM1* | PREDICTED: Homo sapiens G-protein signalling modulator 1 (AGS3-like, C. elegans) (GPSM1), mRNA. | 0.86 | 0.017 |
| NM_001003795.1 | *GTF2IRD2B* | Homo sapiens GTF2I repeat domain containing 2B (GTF2IRD2B), mRNA. | 0.94 | 0.021 |
| NM_012258.2 | *HEY1* | Homo sapiens hairy/enhancer-of-split related with YRPW motif 1 (HEY1), transcript variant 2, mRNA. | 1.06 | 0.036 |
| NM_080596.1 | *HIST1H2AH* | Homo sapiens histone cluster 1, H2ah (HIST1H2AH), mRNA. | 1.03 | 0.048 |
| NM_003516.2 | *HIST2H2AA3* | Homo sapiens histone cluster 2, H2aa3 (HIST2H2AA3), mRNA. | 1.04 | 0.035 |
| NM_176795.2 | *HRAS* | Homo sapiens v-Ha-ras Harvey rat sarcoma viral oncogene homolog (HRAS), transcript variant 1, mRNA. | 0.91 | 0.001 |
| Hs.105575 | *HS.105575* | Homo sapiens mRNA; cDNA DKFZp686C1384 (from clone DKFZp686C1384) | 0.91 | 0.038 |
| Hs.171169 | *HS.171169* | AGENCOURT_13640441 NIH_MGC_186 Homo sapiens cDNA clone IMAGE:30323435 5, mRNA sequence | 1.08 | 0.019 |
| Hs.202577 | *HS.202577* | Homo sapiens cDNA FLJ34585 fis, clone KIDNE2008758 | 1.05 | 0.027 |
| Hs.232649 | *HS.232649* | 602353719F1 NIH_MGC_90 Homo sapiens cDNA clone IMAGE:4451716 5, mRNA sequence | 0.88 | 0.046 |
| Hs.412361 | *HS.412361* | tq42f05.x1 NCI_CGAP_Ut1 Homo sapiens cDNA clone IMAGE:2211489 3, mRNA sequence | 1.13 | 0.006 |
| Hs.532001 | *HS.532001* | ig29e11.y5 HR85 islet Homo sapiens cDNA clone IMAGE:5592692 5, mRNA sequence | 1.10 | 0.026 |
| Hs.571887 | *HS.571887* | Homo sapiens cDNA: FLJ21429 fis, clone COL04205 | 1.05 | 0.018 |
| Hs.95481 | *HS.95481* | tg30c07.x1 NCI_CGAP_Brn25 Homo sapiens cDNA clone IMAGE:2110284 3, mRNA sequence | 0.91 | 0.028 |
| NM_004969.1 | *IDE* | Homo sapiens insulin-degrading enzyme (IDE), mRNA. | 0.86 | 0.027 |
| NM_020748.1 | *INTS2* | Homo sapiens integrator complex subunit 2 (INTS2), mRNA. | 1.04 | 0.049 |
| XM_930788.1 | *KIAA0692* | PREDICTED: Homo sapiens KIAA0692 protein, transcript variant 2 (KIAA0692), mRNA. | 0.96 | 0.001 |
| NM_014949.2 | *KIAA0907* | Homo sapiens KIAA0907 (KIAA0907), mRNA. | 1.07 | 0.033 |
| XM_939177.1 | *KIAA1641* | PREDICTED: Homo sapiens KIAA1641, transcript variant 3 (KIAA1641), mRNA. | 1.10 | 0.027 |
| NM_033060.2 | *KRTAP4-1* | Homo sapiens keratin associated protein 4-1 (KRTAP4-1), mRNA. | 1.05 | 0.013 |
| XR_042310.1 | *LOC100129842* | PREDICTED: Homo sapiens misc_RNA (LOC100129842), miscRNA. | 0.94 | 0.026 |
| XM_001725871.1 | *LOC100130902* | PREDICTED: Homo sapiens hypothetical protein LOC100130902 (LOC100130902), mRNA. | 0.98 | 0.038 |
| XR_037646.1 | *LOC100130914* | PREDICTED: Homo sapiens misc_RNA (LOC100130914), miscRNA. | 1.07 | 0.036 |
| XM_001721907.1 | *LOC100132957* | PREDICTED: Homo sapiens hypothetical protein LOC100132957 (LOC100132957), mRNA. | 1.10 | 0.021 |
| XM_001723587.1 | *LOC100133008* | PREDICTED: Homo sapiens hypothetical protein LOC100133008 (LOC100133008), mRNA. | 1.05 | 0.035 |
| XR_037432.1 | *LOC100133740* | PREDICTED: Homo sapiens misc_RNA (LOC100133740), miscRNA. | 1.05 | 0.040 |
| XR_037714.1 | *LOC400389* | PREDICTED: Homo sapiens misc_RNA (LOC400389), miscRNA. | 0.95 | 0.008 |
| XM_930737.1 | *LOC642351* | PREDICTED: Homo sapiens hypothetical protein LOC642351 (LOC642351), mRNA. | 0.92 | 0.004 |
| XM_928361.1 | *LOC645325* | PREDICTED: Homo sapiens similar to Group X secretory phospholipase A2 precursor (Phosphatidylcholine 2-acylhydrolase GX) (GX sPLA2) (sPLA2-X) (LOC645325), mRNA. | 0.96 | 0.035 |
| XM_928986.1 | *LOC646023* | PREDICTED: Homo sapiens hypothetical protein LOC646023 (LOC646023), mRNA. | 0.94 | 0.042 |
| XM_941325.1 | *LOC647000* | PREDICTED: Homo sapiens similar to tubulin, beta 5 (LOC647000), mRNA. | 0.91 | 0.030 |
| XM_929980.1 | *LOC647000* | PREDICTED: Homo sapiens similar to tubulin, beta 5 (LOC647000), mRNA. | 0.91 | 0.002 |
| XM_937048.1 | *LOC647993* | PREDICTED: Homo sapiens hypothetical protein LOC647993 (LOC647993), mRNA. | 0.95 | 0.008 |
| XM_941346.1 | *LOC652038* | PREDICTED: Homo sapiens hypothetical protein LOC652038 (LOC652038), mRNA. | 1.13 | 0.029 |
| XR_038223.1 | *LOC728820* | PREDICTED: Homo sapiens misc_RNA (LOC728820), miscRNA. | 1.05 | 0.042 |
| XM_001130675.1 | *LOC729570* | PREDICTED: Homo sapiens similar to hCG1793303 (LOC729570), mRNA. | 0.90 | 0.001 |
| NR_015341.1 | *LRRC37B2* | Homo sapiens leucine rich repeat containing 37, member B2 (LRRC37B2), non-coding RNA. | 1.35 | 0.045 |
| NM_144586.3 | *LYPD1* | Homo sapiens LY6/PLAUR domain containing 1 (LYPD1), transcript variant 1, mRNA. | 1.12 | 0.010 |
| NM_001003897.1 | *MANBAL* | Homo sapiens mannosidase, beta A, lysosomal-like (MANBAL), transcript variant 2, mRNA. | 0.88 | 0.020 |
| NM_033412.2 | *MCART1* | Homo sapiens mitochondrial carrier triple repeat 1 (MCART1), nuclear gene encoding mitochondrial protein, mRNA. | 0.93 | 0.010 |
| NM_015246.1 | *MGRN1* | Homo sapiens mahogunin, ring finger 1 (MGRN1), mRNA. | 1.23 | 0.022 |
| NR_030227.1 | *MIR450A2* | Homo sapiens microRNA 450a-2 (MIR450A2), microRNA. | 1.08 | 0.021 |
| NM_145644.1 | *MRPL35* | Homo sapiens mitochondrial ribosomal protein L35 (MRPL35), nuclear gene encoding mitochondrial protein, transcript variant 2, mRNA. | 0.93 | 0.044 |
| NM_021107.1 | *MRPS12* | Homo sapiens mitochondrial ribosomal protein S12 (MRPS12), nuclear gene encoding mitochondrial protein, transcript variant 1, mRNA. | 1.07 | 0.046 |
| NM_033111.3 | *N4BP2L2* | Homo sapiens NEDD4 binding protein 2-like 2 (N4BP2L2), transcript variant 1, mRNA. | 1.08 | 0.043 |
| NM_024608.1 | *NEIL1* | Homo sapiens nei endonuclease VIII-like 1 (E. coli) (NEIL1), mRNA. | 0.94 | 0.006 |
| NM_001007595.1 | *NLF2* | PREDICTED: Homo sapiens nuclear localized factor 2 (NLF2), mRNA. | 0.96 | 0.010 |
| NM_016391.4 | *NOP16* | Homo sapiens NOP16 nucleolar protein homolog (yeast) (NOP16), mRNA. | 0.88 | 0.030 |
| XM_936449.1 | *P2RX2* | PREDICTED: Homo sapiens purinergic receptor P2X, ligand-gated ion channel, 2 (P2RX2), mRNA. | 0.96 | 0.030 |
| NM_020367.3 | *PARP11* | Homo sapiens poly (ADP-ribose) polymerase family, member 11 (PARP11), mRNA. | 0.92 | 0.019 |
| NM_031268.4 | *PDPK1* | Homo sapiens 3-phosphoinositide dependent protein kinase-1 (PDPK1), transcript variant 2, mRNA. | 1.13 | 0.015 |
| NM_181775.3 | *PLXNA4* | Homo sapiens plexin A4 (PLXNA4), transcript variant 2, mRNA. | 1.17 | 0.010 |
| NM_004582.2 | *RABGGTB* | Homo sapiens Rab geranylgeranyltransferase, beta subunit (RABGGTB), mRNA. | 0.90 | 0.042 |
| NM_019027.3 | *RBM47* | Homo sapiens RNA binding motif protein 47 (RBM47), transcript variant 2, mRNA. | 0.93 | 0.017 |
| NM_001098634.1 | *RBM47* | Homo sapiens RNA binding motif protein 47 (RBM47), transcript variant 1, mRNA. | 0.90 | 0.046 |
| NM_018715.1 | *RCC2* | Homo sapiens regulator of chromosome condensation 2 (RCC2), mRNA. | 0.89 | 0.019 |
| NM_134427.1 | *RGS3* | Homo sapiens regulator of G-protein signaling 3 (RGS3), transcript variant 4, mRNA. | 1.07 | 0.017 |
| NM_000980.2 | *RPL18A* | Homo sapiens ribosomal protein L18a (RPL18A), mRNA. | 0.94 | 0.046 |
| NM_000969.3 | *RPL5* | Homo sapiens ribosomal protein L5 (RPL5), mRNA. | 0.83 | 0.019 |
| NM_003028.1 | *SHB* | Homo sapiens Src homology 2 domain containing adaptor protein B (SHB), mRNA. | 0.94 | 0.034 |
| NM_194298.1 | *SLC16A9* | Homo sapiens solute carrier family 16, member 9 (monocarboxylic acid transporter 9) (SLC16A9), mRNA. | 0.97 | 0.006 |
| NM_005116.5 | *SLC23A2* | Homo sapiens solute carrier family 23 (nucleobase transporters), member 2 (SLC23A2), transcript variant 1, mRNA. | 1.13 | 0.034 |
| NM_033518.1 | *SLC38A5* | Homo sapiens solute carrier family 38, member 5 (SLC38A5), mRNA. | 1.08 | 0.023 |
| NM_014011.4 | *SOCS5* | Homo sapiens suppressor of cytokine signaling 5 (SOCS5), transcript variant 1, mRNA. | 1.03 | 0.028 |
| NM_003116.1 | *SPAG4* | Homo sapiens sperm associated antigen 4 (SPAG4), mRNA. | 0.97 | 0.015 |
| NM_003198.1 | *TCEB3* | Homo sapiens transcription elongation factor B (SIII), polypeptide 3 (110kDa, elongin A) (TCEB3), mRNA. | 0.95 | 0.045 |
| NM_030625.2 | *TET1* | Homo sapiens tet oncogene 1 (TET1), mRNA. | 1.04 | 0.028 |
| NM_015963.4 | *THAP4* | Homo sapiens THAP domain containing 4 (THAP4), mRNA. | 0.94 | 0.049 |
| NM_182578.1 | *THEM5* | Homo sapiens thioesterase superfamily member 5 (THEM5), mRNA. | 1.07 | 0.001 |
| NM_021210.2 | *TRAPPC1* | Homo sapiens trafficking protein particle complex 1 (TRAPPC1), mRNA. | 0.93 | 0.023 |
| NM_178014.2 | *TUBB* | Homo sapiens tubulin, beta (TUBB), mRNA. | 0.90 | 0.027 |
| NM_181762.1 | *UBE2A* | Homo sapiens ubiquitin-conjugating enzyme E2A (RAD6 homolog) (UBE2A), transcript variant 2, mRNA. | 0.88 | 0.050 |
| NM_006786.2 | *UTS2* | Homo sapiens urotensin 2 (UTS2), transcript variant 2, mRNA. | 1.06 | 0.038 |
| NR_024540.1 | *WASH5P* | Homo sapiens WAS protein family homolog 5 pseudogene (WASH5P), non-coding RNA. | 1.05 | 0.044 |
| NM_138458.1 | *WDR92* | Homo sapiens WD repeat domain 92 (WDR92), mRNA. | 0.92 | 0.024 |
| NM_025189.2 | *ZNF430* | Homo sapiens zinc finger protein 430 (ZNF430), mRNA. | 1.13 | 0.010 |
| NM_001023563.1 | *ZNF805* | Homo sapiens zinc finger protein 805 (ZNF805), mRNA. | 1.04 | 0.022 |
